# Alterations of the Intracellular Peptidome in Response to the Proteasome Inhibitor Bortezomib

**DOI:** 10.1371/journal.pone.0053263

**Published:** 2013-01-07

**Authors:** Julia S. Gelman, Juan Sironi, Iryna Berezniuk, Sayani Dasgupta, Leandro M. Castro, Fabio C. Gozzo, Emer S. Ferro, Lloyd D. Fricker

**Affiliations:** 1 Department of Molecular Pharmacology, Albert Einstein College of Medicine, Bronx, New York, United States of America; 2 Department of Neuroscience, Albert Einstein College of Medicine, Bronx, New York, United States of America; 3 Department of Cell Biology and Development, University of São Paulo, São Paulo, Brazil; 4 Chemistry Institute, State University of Campinas, São Paulo, Brazil; University of Illinois at Chicago, United States of America

## Abstract

Bortezomib is an antitumor drug that competitively inhibits proteasome beta-1 and beta-5 subunits. While the impact of bortezomib on protein stability is known, the effect of this drug on intracellular peptides has not been previously explored. A quantitative peptidomics technique was used to examine the effect of treating human embryonic kidney 293T (HEK293T) cells with 5–500 nM bortezomib for various lengths of time (30 minutes to 16 hours), and human neuroblastoma SH-SY5Y cells with 500 nM bortezomib for 1 hour. Although bortezomib treatment decreased the levels of some intracellular peptides, the majority of peptides were increased by 50–500 nM bortezomib. Peptides requiring cleavage at acidic and hydrophobic sites, which involve beta-1 and -5 proteasome subunits, were among those elevated by bortezomib. In contrast, the proteasome inhibitor epoxomicin caused a decrease in the levels of many of these peptides. Although bortezomib can induce autophagy under certain conditions, the rapid bortezomib-mediated increase in peptide levels did not correlate with the induction of autophagy. Taken together, the present data indicate that bortezomib alters the balance of intracellular peptides, which may contribute to the biological effects of this drug.

## Introduction

Peptidomic studies have identified hundreds of intracellular peptides derived from cytosolic, mitochondrial, and nuclear proteins in mammalian cells [1]–[6]. It is possible that some of these naturally-occurring intracellular peptides modulate cellular activities based on the finding that synthetic peptides of 10–20 amino acids can mimic or block protein functions and produce physiological changes in cellular function [7]–[9]. In some cases, the synthetic peptides used to produce cellular changes corresponded to peptides found in peptidomics analyses of the tissue. For example, the addition of specific peptides was shown to modulate the signal transduction elicited by agonists of G-protein coupled receptors in HEK293 and CHO cells [9]. Intracellular peptides derived from rat adipose tissue proteins facilitate insulin-induced glucose uptake in 3T3-L1 adipocytes [10]. In *C.elegans*, peptides produced from mitochondrial proteins were shown to signal nuclear-encoded mitochondrial chaperone genes and indicate the stress of mitochondrial protein misfolding [11]. In *Drosophila*, peptides encoded by small open reading frame genes were found to control epidermal differentiation by modifying the activity of transcription factors [12]. Thus, an emerging concept is that peptides produced from cytosolic, mitochondrial, and/or nuclear proteins have functional roles in cellular processes, and are not merely intermediates in the protein degradation pathway [13].

There are four major peptide-generating systems within cells: proteasomes, calpains, caspases, and lysosomes [14]–[17]. The proteasome complex plays a major role in protein turnover, degrading proteins into peptides of 4–25 amino acids with an average size around 10 amino acids [14]. Calpains are a family of calcium-regulated proteases that perform limited proteolysis [15]. Caspases are also a family of intracellular proteases, but with a strict substrate specificity for cleavage at sites containing an Asp residue [18]. Lysosomes are organelles that degrade proteins by a series of endo- and exopeptidase activities [17]. In addition to these proteases, a number of cytosolic oligopeptidases exist, including thimet oligopeptidase (endopeptidase 24.15), neurolysin (endopeptidase 24.16), post-prolyl oligopeptidase, nardilysin, and insulin degrading enzyme [19]–[25]. These oligopeptidases are not capable of cleaving proteins; they selectively cleave a subset of cellular peptides into smaller fragments [19]–[24], [26]. Degradation of intracellular peptides into amino acids occurs through the action of aminopeptidases and other enzymes [27], [28].

Previous studies aimed at determining the proteolytic system involved in producing the intracellular peptides of human embryonic kidney 293T (HEK293T) cells implicated the proteasome complex and not calpains based on the observation that epoxomicin (a proteasome inhibitor) but not A23187 (a calcium ionophore) affected intracellular peptide levels [29], [30]. Epoxomicin is an irreversible inhibitor of the proteasome, potently inhibiting the beta-5 subunit (which cleaves proteins at hydrophobic amino acids) and less potently inhibiting the beta-2 subunit (which cleaves proteins at basic amino acids) [31]. Consistent with this activity of epoxomicin, most of the intracellular peptides that resulted from protein cleavage at hydrophobic sites were greatly reduced by 0.2 µM epoxomicin while those peptides that resulted from protein cleavage at basic amino acids were reduced by 2 µM epoxomicin but not by 0.2 µM epoxomicin [29]. Furthermore, many of the intracellular peptides that resulted from cleavage at beta-1 sites (acidic amino acids) were elevated by epoxomicin treatment; this is consistent with the idea that proteins transported into the epoxomicin-inhibited proteasome cannot be cleaved at their normal sites (i.e. beta-2 or -5) and as a result there is increased activity at alternate sites (i.e. beta-1).

Bortezomib has been reported to be a highly selective proteasome inhibitor with greatest potency for the beta-5 subunit and lower potency for the beta-1 subunit [32]–[34]. Bortezomib has been successful for the treatment of several types of cancer, including multiple myeloma [35]–[37]. A major side effect of bortezomib is neuropathy, presumably due to the action of the drug on nerve cells. In the present study, we tested the effect of bortezomib on levels of peptides in two different cell lines that have been extensively used in previous peptidomic studies: HEK293T and human neuroblastoma-derived SH-SY5Y cells. Cells were treated with a sub-toxic level of bortezomib for 1, 6, or 16 hours, or with higher concentrations for 30, 60, or 90 minutes, and then the peptidome examined using a quantitative peptidomics approach [5], [38]. Levels of some peptides were reduced by treatment with bortezomib, consistent with the hypothesis that the proteasome produces these peptides. However, many other peptides were elevated by bortezomib treatment, including a large number that contained hydrophobic residues in the cleavage sites. This raises the possibility that bortezomib affects the cellular peptidome by changing the processing pathways. The global change in peptide levels caused by bortezomib may contribute to the physiological effects of this important anticancer drug.

## Materials and Methods

### Reagents

Hydroxylamine, glycine, sodium hydroxide, sodium phosphate, 3-(4, 5-dimethylthiazol-2-yl)-2,5-diphenyl tetrazolium bromide (MTT), and dimethyl sulfoxide (DMSO), were obtained from Sigma. Acetonitrile was obtained from Fisher. Hydrochloric acid and trifluoroacetic acid mass spectroscopy grade were obtained from Pierce Thermo Scientific. Bortezomib was obtained from LC Laboratories (Woburn, MA). The isotopic labeling reagents 4-trimethylammoniumbutyryl-N-hydroxysuccinimide (TMAB-NHS) containing either 0, 3, 6, or 9 atoms of deuterium (D0-, D3-, D6-, and D9-, respectively) or 9 atoms of deuterium and three ^13^C atoms (D12-) were synthesized as described [39].

### Cell Viability Assay

Cellular sensitivity was determined using MTT, as described [40]. In brief, HEK293T cells were plated in 12 or 48 well plates at 20–30% confluency the day before treatment with bortezomib. The cells were treated with different concentrations of bortezomib for 16 hrs. At the end of the incubation time the medium was replaced with PBS containing 5% fetal bovine serum and 0.5 mg/ml of MTT. After 2 hrs incubation the media were removed and the formazan crystals were dissolved with 0.1 N HCl in anhydrous isopropanol in an amount equal to the original culture volume. The absorbance was measured at 570 nm, and the background at 690 nm subtracted. Cell death was also monitored by light microscopy and morphological changes were observed in correlation with the bortezomib treatment in agreement with the MTT values (data not shown).

Cells were disrupted by pipetting in lysis buffer (50 mM Tris-HCl, pH 8.0, 120 mM NaCl, 0.5% Nonidet P-40, 100 mM NaF, 200 µM sodium orthovanadate, and protease inhibitors) and analyzed on a denaturing polyacrylamide gel (SDS-PAGE). Protein concentration was determined by Bradford assay (Bio-Rad). Total lysates were separated by SDS-PAGE on 4–15% gradient gels (Bio-Rad) and stained with Coomassie blue.

### Large Scale Cell Culture, Bortezomib Treatment, and Peptide Extraction

HEK293T and SH-SY5Y cells were grown to 90% confluence in 15 cm cell culture plates in high glucose Dulbecco’s Modified Eagle’s Medium (D-MEM, Invitrogen 11995), supplemented with 10% fetal bovine serum and pen/strep antibiotic. Two to three plates of cells were used per group. At the start of the experiment, media were removed from all plates and replaced either with media containing bortezomib (5 nM, 50 nM, or 500 nM, diluted from a 10 mM stock in DMSO) or containing the same amount of DMSO (0.025% or less, depending on dilution) as controls. Plates were incubated at 37°C for 0.5, 1.0, 1.5, 6, or 16 hrs. Each concentration of drug and length of incubation included two or three treated groups of cells and two control groups, as shown in Figure S1 (in Supplement). For the 16 hr time point with 5 nM bortezomib, the entire experiment was repeated to provide 6 biological replicates and 4 control groups.

Following incubation, cells were washed three times with Dulbecco’s phosphate-buffered saline (DPBS, Invitrogen) and centrifuged at 800×g for 5 min. In some experiments, bortezomib was included in the DPBS at the same concentration as used for the incubation. The cell pellet was resuspended 1 ml of 80°C water, the mixture was incubated for 20 min in an 80°C water bath and then cooled and transferred to a 2 ml low retention microfuge tube for centrifugation (4°C, 13,000×g). The samples were then stored at −70°C overnight. For peptide extraction, the samples were thawed and centrifuged again. The supernatant was collected and concentrated in a vacuum centrifuge to ∼750 µL. The samples were cooled on ice and acidified with 0.1 M HCl (∼75 µL) to a final concentration of 10 mM HCl. Following 15 minute incubation on ice, the samples were centrifuged (13,000×g for 40 min at 4°C) and the supernatants stored at −70°C until labeling.

### Isotopic Labeling and Mass Spectrometry

The labeling procedure has been described previously [39]. Briefly, each group within an experiment was labeled with a different isotopic TMAB-NHS label. A typical labeling scheme is shown in Figure S1; in this scheme, the three biological replicates of the bortezomib-treated cells were labeled with D0-, D6-, and D12-TMAB-NHS while the two biological replicates of the control groups were labeled with D3- and D9-TMAB-NHS. This labeling scheme was altered between experiments; some bortezomib-treated samples were labeled with D0-, D3, and D6-TMAB-NHS, while in other experiments the bortezomib-treated groups were labeled with D3-, D6-, and D9-TMAB-NHS.

To label the peptides, 250 µL of 0.4 M phosphate buffer (pH 9.5) was added to peptide extracts and the pH of the samples was adjusted to 9.5 using 1 M NaOH. Labeling was done in 7 rounds. Solutions of labels were initially prepared (dissolved in DMSO), using 7.5 mg of TMAB-NHS label per 15 cm plate of cells, and 1/7^th^ of this solution was added to the peptide every 20 minutes. Between each round of adding labels, the pH was checked and adjusted back to 9.5 with 1M NaOH if necessary. After all 7 rounds of label addition were complete, the pH was again brought to 9.5 and samples were incubated at room temperature for 60 minutes, after which 30 µL of 2.5 M glycine was added in order to quench any remaining labeling reagent. Following 40 minute incubation at room temperature, the samples labeled with different isotopic tags were pooled, concentrated to 2 ml and filtered through a Millipore Microcon YM-10 unit. In order to remove any TMAB labels on Tyrosine residues, hydroxylamine was added in three rounds for a total of 5 µL of 2.0 M hydroxylamine per 15 mg TMAB label. The pH was adjusted to 9.0 before each addition of hydroxylamine. The last step of the process was to desalt the samples using C18 spin columns (Pierce-Thermo). The process of desalting was done as described by the manufacturer. Peptides were eluted from the C18 column with 160 µL of 0.5% trifluoroacetic acid in 70% acetonitrile. The eluate was dried in a vacuum centrifuge and stored at −70°C until further analysis.

The labeled peptides were analyzed by liquid chromatography/mass spectrometry (LC-MS/MS) on a Synapt G1 mass spectrometer (Waters Co., EUA). The peptide mixture was resuspended in 10 µl of water and 2–5 µl injected onto a Symmetry C18 trapping column (5 µm particles, 180 µm i.d.×20 mm, Waters, USA). The samples were desalted for 15 min and the trapped peptides were then separated by elution with a water/acetonitrile 0.1% formic acid gradient through a BEH 130 - C18 column (1.7 µm particles, 100 µm i.d. ×100 mm, Waters, USA), as previously described [26]. Data were acquired in data-dependent mode and selected peptides dissociated by collisions with argon. The liquid chromatography and electrospray ionization conditions included a flow rate of 600 nL/min, nanoflow capillary voltage of 3.5 kV, block temperature of 100°C, and cone voltage of 100 V.

The MS spectra were analyzed using the MassLynx software (Waters) to identify groups of peaks representing peptides labeled with the different isotope tags (see Figure S1 for representative data). Quantification was performed by first determining the relative intensity of each isotopic peak, considering both the mono-isotopic and the peak containing one atom of ^13^C and subtracting the baseline due to lower-mass peaks (Figure S1). The intensity of each of the 4–5 isotopic TMAB-labeled peptides was compared to the average of the two control replicates in each group.

To identify the peptides, the MS/MS data was analyzed using the Mascot search engine (Matrix Science Ltd, UK) and the IPI_human data base (91,464 sequences; 36,355,611 residues) with variable modifications of N-terminal acetylation, methionine oxidation, and the isotopic D0-, D3-, D6-, and D9-TMAB tags used in our study (listed on Mascot as GIST-Quat). The D12-TMAB tag used for one of the replicates in our study is not available as a search option in Mascot. Results were manually interpreted to eliminate false positives, using criteria previously described [26], [39], [41], [42]. In brief, these criteria include the following nine points: (i) the isotopic form of TMAB selected by Mascot corresponds to the isotopic form based on analysis of the peak set. Mascot does not recognize which of the individual peaks correspond to the D0, D3, D6, D9, or D12 forms, and therefore the correlation of the isotopic TMAB form in the observed peak set with the predicted Mascot match is a simple and necessary step that removes the majority of false positives. (ii) The number of tags found to be incorporated into the peptide (based on the mass difference between peaks) matches the number of free amines in the peptide (i.e. the side chain amines of Lys and the N-terminus, if not blocked by modification). For those peptides that incorporate multiple tags (due to more than one free amine), all tags must be the same isotopic form on a particular peptide. False positives will rarely have consistent forms of the tags, while true positives must always have the same isotopic form of the tag. (iii) The Mascot score is either the top score of all potential peptides, or the other peptides with comparable scores can be excluded by the above criteria. (iv) The vast majority of the observed MS/MS fragment ions match predicted a, b, or y ions, internal ions, or precursor ions with loss of trimethylamine. (v) At least five fragment ions match b- or y-series ions. (vi) The mass accuracy of the fragment ions is within the accepted specification for the q-TOF instrument used for the analysis, usually within 20 parts per million. (vii) The observed charge state of the peptide should match the predicted charge state, usually corresponding to the number of isotopic tags (which are positively charged) plus the number of Arg and His residues, although His residues are not always positively charged and often exist with two different charge states. Some peptides pick up an additional proton to give a charge state one higher than the maximum predicted from the number of amines, and some lose a proton (from –COOH groups on Glu, Asp, and the C-terminus) to give a charge state lower than expected. However, the ion with the correct charge state is always found. (viii) The fragment ions match the expected ions based on the peptide sequence. For example, fragmentation of Xaa-Pro bonds is favored, while fragmentation of Pro-Xaa bonds is rarely observed [43]. The b2 and a2 ions are usually very strong if the N-terminus has a TMAB-label attached, unless there is an N-terminal Pro or a basic residue in positions 1 or 2. (ix) For some peptides, MS/MS fragmentation was not obtained in a particular LC/MS run performed for the samples in the present study, but the peptide was identified by MS/MS analysis of another run, using the above criteria to validate the Mascot results. In these cases, the peptide was considered to be identical if the observed mass was within 40 ppm of the theoretical value and the number of TMAB tags, charge state, and elute time from the LC column were comparable to the peptide identified by MS/MS of another run.

## Results

**Figure 1:**
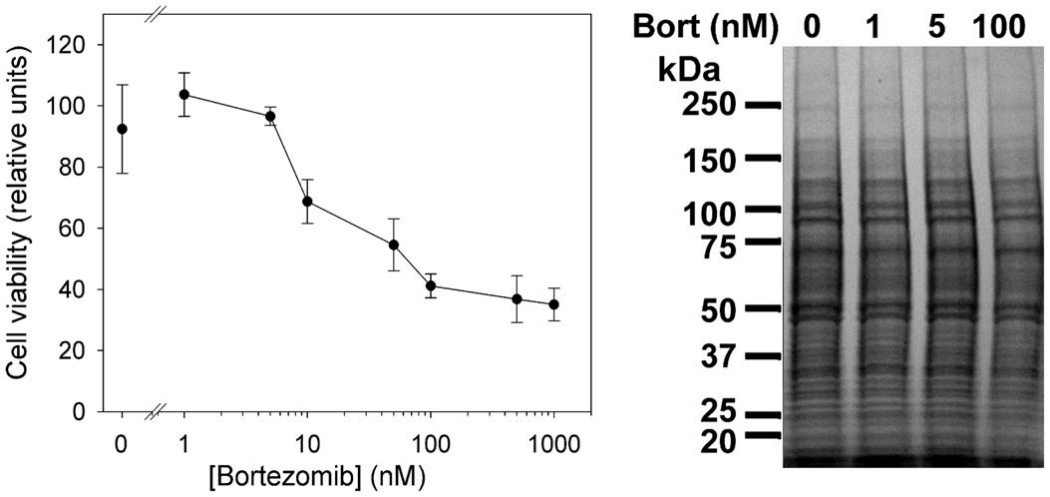
Dose response of HEK293T cells to bortezomib. *Left*: Cell viability profile of HEK293T cells treated with different concentrations of bortezomib for 16 hrs and quantified by MTT. The values represent the average of four replicates; error bars show standard error of the mean. *Right*: Coomassie blue stained gel electrophoresis of cell lysates untreated (0) or treated with 1, 5, or 100 nM of bortezomib. Similar volumes of cells lysates representing equal amount of starting number of cells (before treatment) were loaded.

Bortezomib has been tested in a variety of cell lines, and typically has shown effects in the nM concentration range [44]. Therefore, we first tested a range of concentrations with the HEK293T cell line in order to determine the highest level of bortezomib that would not cause substantial cell death during a 16 hr incubation. Mitochondrial function was assayed using MTT; this reflects overall cell viability [40]. Concentrations of bortezomib above 10 nM caused substantial cell death over the 16 h incubation (Figure 1). Bortezomib at a concentration of 5 nM showed cell viability comparable to untreated cells. Therefore, experiments involving long-term treatment used this concentration of bortezomib. Short-term treatments also used 5 nM bortezomib as well as 50 and 500 nM; these higher concentrations have previously been found to substantially inhibit proteasome activity in various cell lines [32]–[34], [45]. The proteasome activity of HEK293T cell extracts was significantly inhibited by 5, 50, and 500 nM bortezomib (Supplemental Figure S2). In addition to the studies on HEK293T cells, we also tested a neuroblastoma cell line (SH-SY5Y cells) with 500 nM bortezomib for 1 hour; SH-SY5Y cells were previously examined for peptide content and found to have many of the same peptides as found in HEK293T cells [30]. We also tested a short incubation of HEK293T cells with 500 nM bortezomib in which the drug was included in the PBS washes (total incubation time 30 minutes with drug), and also a longer treatment time with 500 nM bortezomib in which the drug was included in the PBS washes (total incubation time 90 minutes with drug).

Quantification of the relative levels of peptide in the bortezomib-treated and control cells was performed by measurement of peak intensity for each of the isotopic peaks; for this analysis, both the monoisotopic peak and the peak with one ^13^C atom were considered (Figure S1). In the present study, a total of 310 distinct peptides were identified by MS/MS sequence analysis. Approximately 70% of these were previously found in other peptidomics studies of HEK293T and SH-SY5Y cells [26], [30], while the other 30% are newly identified. In addition to the identified peptides, over 500 additional peptides were detected in one or more of the various LC/MS runs performed in the present study. Many of the peptides were found in multiple LC/MS runs representing different concentrations or treatment times. The entire data set is shown in Table S1 (supplement). The ratio of the peptide level in each group relative to the average control level for that experiment is indicated in Table S1.

**Figure 2:**
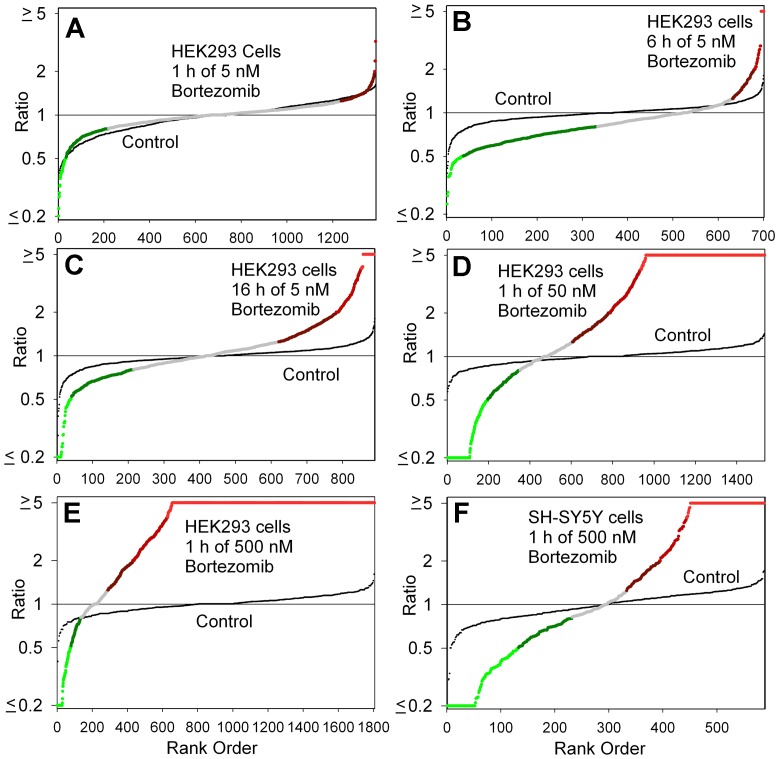
Summary plots of peptidomic analysis. The level of peptide in each biological replicate, relative to the average control level, was determined for all peptides detected in the analysis of the MS data. For this analysis, if the ratio was <0.20 or >5.0 between the drug-treated samples and the average control, it was capped at 0.20 or 5.0. The ratios for all detected peptides in each experiment were then sorted from low to high and the result plotted. The y-axis represents the observed ratio (log scale) and the x-axis represents each individual replicate. In each panel, two plots are shown: one plot shows the differences between the bortezomib-treated samples and the average control value (large colored circles), the other plot shows the relative level of each control replicate compared to the average control value (small black circles). For the bortezomib-treated samples, the color of the circle corresponds to the color scheme used in figures 3 and 5. Panels A-E show data from experiments on HEK293T cells; Panel F is for SH-SY5Y cells. Panels A-C tested 5 nM bortezomib for either 1 hour (A), 6 hours (B), or 16 hours (C). Panels D-E examined 1 hour time points with either 50 nM bortezomib (D) or 500 nM bortezomib (E, F).

A variety of analyses were performed on the data. To visualize the effect of a treatment on overall peptide levels, the level of each peptide in a replicate was divided by the average control level of that peptide, and the ratio for each peptide and replicate was combined, sorted from low to high, and plotted (Figure 2). For these plots, the x-axis represents the peptide rank when the relative levels are sorted from low to high, and the y-axis represents the relative level (with the lowest ratio capped at 0.20 for those peptides with values below this, and the highest ratio capped at 5.0 for peptides with a larger increase). In each plot, the variation of the individual control groups, relative to the average control, is plotted as small black circles. The large colored circles represent the groups of bortezomib-treated cells, relative to the average control groups. The color of the circle represents the same color scheme used in subsequent figures; peptides that show a large decrease relative to the average control (ratio ≤0.50) are in bright green; peptides that show a moderate decrease (0.51 to 0.80) are in dark green; peptides not greatly altered (0.81 to 1.24) are in grey; peptides slightly elevated (1.25 to 1.99) are in dark red; peptides showing a greater increase (2.00 to 3.99) are in medium-bright red, and peptides greatly increased (≥4.0) are in bright red. From these plots, it is apparent that treatment of HEK293T cells with 5 nM bortezomib for 1 hour does not change the levels of most peptides (Figure 2A). In contrast, incubating longer with the same concentration of bortezomib (Figure 2B-C) or for the same time with higher concentrations (Figure 2D-E) causes a change in the level of many peptides. As expected, the bortezomib treatment led to a decrease in the levels of some peptides, consistent with the hypothesis that the proteasome was responsible for the generation of many of the observed cellular peptides. However, an unexpected finding was that a very large number of peptides were elevated by the bortezomib treatment, especially when HEK293T cells were incubated with 500 nM of the drug for 1 hour (Figure 2E). A second cell line, the human neuroblastoma SH-SY5Y cell line was also treated with 500 nM bortezomib for 1 hour and examined by peptidomics, and an increase in many peptides was also detected (Figure 2F). The HEK293T cells incubated with 500 nM bortezomib for a total time of 30 or 90 minutes showed changes similar to those observed with the 60 minute treatment (see Table S1 for data).

**Figure 3:**
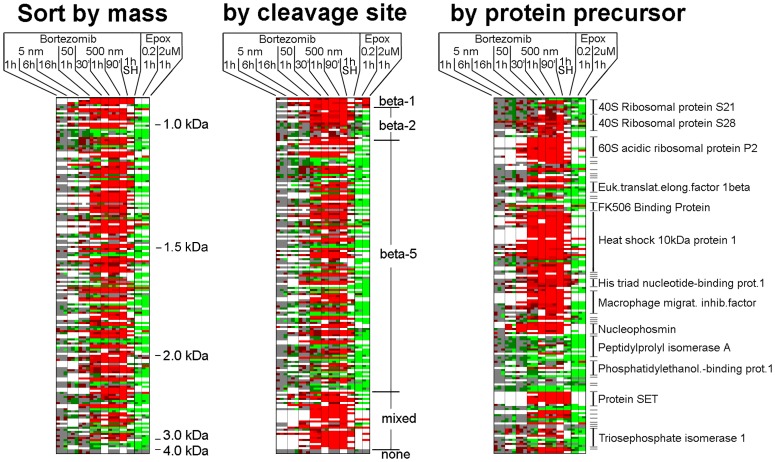
Heat map analysis of selected peptides. Peptides chosen for this analysis were observed in at least 3 separate experiments. The biological replicates within each experiment are indicated as separate sub-columns, and the color indicates the ratio (using the same color scheme as defined in Figure 2). White panels could not be quantified, either due to undetectable signals or peak overlap with another co-eluting peptide. All data are for HEK293T cells except for the one experiment performed with SH-SY5Y cells (SH). The data from the treatment of HEK293T cells with epoxomicin (either 0.2 or 2 µM) were previously published [29], although these data were reanalyzed to screen for additional peptides identified in the present study with bortezomib. The data used for the heat map are shown in supplemental Table S2, which includes peptide identity, protein name, ratio values, and other information.

The analysis shown in Figure 2 represents every group of cells within each experiment, and does not provide information on the variability of a peptide within the replicates of each experiment or between experiments. For this, we analyzed the data using a heat map-type plot (Figure 3 and Table S2). The color scheme used in the heat map is identical to the color scheme used in Figure 2, with white squares representing missing data (due to peak overlap or undetectable signals). Only peptides found in three or more experiments were included in the heat map, for a total of 173 peptides. Each of the 25 columns represents a separate group of cells within the various experiments (2–3 replicates for HEK293T cells, 2 replicates for SH-SY5Y cells). For the majority of peptides, the relative level of peptide in the replicates of bortezomib-treated cells was fairly close and the resulting color of the squares was either the same for all replicates, or reflected minor differences that caused one of the replicates to be in a different bin than the other replicates. In addition to showing that the results from the replicates in each experiment are generally close, the heat map shows that many of the peptides altered by bortezomib in one experiment are similarly affected in other experiments (Figure 3). For example, most of the peptides altered by treatment of HEK293T cells with 500 nM bortezomib for 1 hour are similarly affected in HEK293T cells treated with 500 nM bortezomib for 30 or 90 minutes, or with 50 nM bortezomib for 1 hour. The SH-SY5Y cell line treated with 500 nM bortezomib for 1 hour also showed many of the same changes, although the peptidomes of the different cell lines were not identical, as previously noted [30]. The long-term treatment of HEK293T cells with 5 nM bortezomib caused some of the same changes as those observed with higher concentrations for shorter time periods (Figure 3).

The heat map analysis was sorted by different parameters. When sorted by peptide mass, there is no clear correlation between the changes in peptide levels and peptide size (Figure 3, left panel). When sorted by cleavage site, all peptides that arise from cleavage at acidic residues (i.e. beta-1 sites) and most of the peptides that require cleavage at basic residues (i.e. beta-2 sites) are elevated by bortezomib treatment (Figure 3, middle panel). In addition, many of the peptides that are generated by cleavage at hydrophobic residues (i.e. beta-5 sites) are also elevated by the bortezomib treatment. This finding is much different than the results with epoxomicin, a proteasome inhibitor that also shows greatest potency towards the beta-5 site, and which generally causes a decrease in levels of peptides that require cleavage at beta-5 sites [29]. Because our previous data with epoxomicin did not include all of the peptides found in the present study, we re-analyzed the epoxomicin data to search for every peptide found in the present study; those that were found are included together with the previously published epoxomicin data in the heat maps in Figure 3 (and included in Supplementary Table S2). Although both bortezomib and epoxomicin are potent inhibitors of the beta-5 proteasome subunit, there are dozens of likely beta-5 products that are decreased by epoxomicin treatment but elevated by bortezomib treatment (Figure 3). Sorting the heat map data by protein precursor reveals that many of the peptides elevated by bortezomib treatment arise from just six proteins: 60S acidic ribosomal protein P2, heat shock 10kDa protein 1, histidine triade nucleotide-binding protein 1, macrophage migration inhibitory factor, nucleophosmin, and protein SET (Figure 3, right panel). Collectively, these six proteins account for 65 of the peptides that are greatly elevated upon bortezomib treatment.

**Figure 4:**
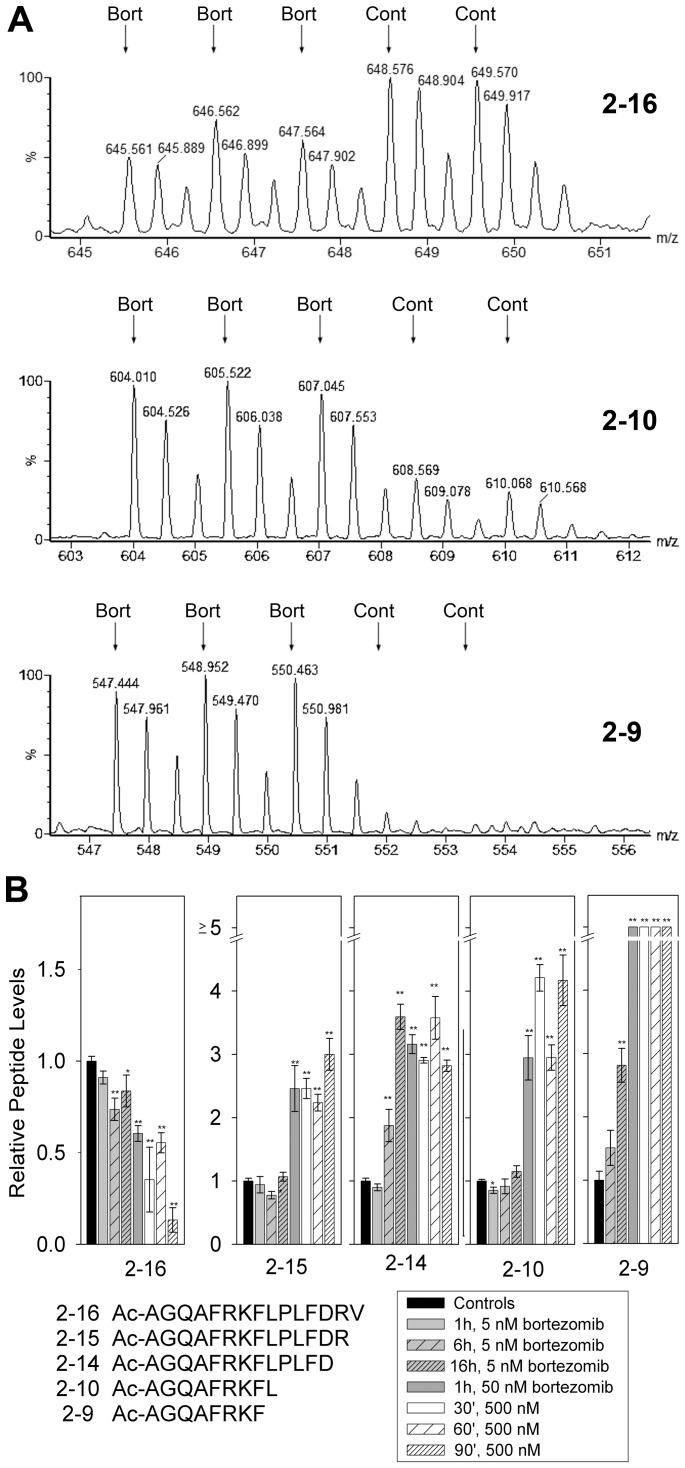
Analysis of the N-terminal fragments of heat shock 10kDa protein 1 (chaperonin 10) upon bortezomib treatment. A: Representative MS data from the experiment testing HEK293T cells with 500 nM bortezomib for 1 hour. In this experiment the three bortezomib-treated replicates were labeled with D0-, D3-, and D6-TMAB-NHS and the control replicates were labeled with D9- and D12-TMAB-NHS. Mass spectra are shown for the N-terminal fragments representing residues 2–16, 2–10, and 2–9 of heat shock 10kDa protein 1 (chaperonin 10). B: Relative levels of the three peptides shown in Panel A, and of 2 additional peptides in the various experiments. Error bars show standard error of the mean for 2–3 replicates for the bortezomib-treated samples (see Table S1 for original data and number of replicates). For the controls, data from each of the experiments were pooled, resulting in n values of 8–10. *, p<0.05; **, p<0.01 versus control using Student’s t-test.

Of all proteins listed in Figure 3, heat shock 10 kDa protein 1 (also known as chaperonin 10) gave rise to more peptides than any other protein detected in the present study. Five of these heat shock 10kDa protein 1-derived peptides represented different N-terminal fragments, allowing for a direct comparison of the impact of bortezomib on cleavage site. The longest of these peptides, residues 2–16 of the protein, showed a consistent decrease in all three replicates of the cells treated with 500 nM bortezomib for 1 hour (Figure 4A). This decrease was statistically significant (p<0.01 using Student’s t-test). Significant decreases in this peptide were also observed in the experiments testing 5 nM bortezomib for 6 and 16 hrs, with 50 nM drug for 1 hour, and with 500 nM drug for 30 and 90 minutes (Figure 4B). Because this peptide represents the N-terminus of the protein, a single cleavage is sufficient to generate the peptide. The P1 residue of the cleavage site is a Val, and this would be expected to be cleaved by the β5-proteasome subunit; the decrease in levels of this peptide upon bortezomib treatment is consistent with a role for the proteasome in the production of this peptide. The shorter N-terminal fragment corresponding to position 2–15 is slightly elevated by the treatment with 50 or 500 nM bortezomib for all time points examined (Figure 4B). This peptide has a C-terminal Arg residue, and therefore represents cleavage by a β2-like proteasome subunit. Because this subunit is not inhibited by any of the concentrations of bortezomib used in the present study, the resulting peptide would not be expected to decrease in the presence of the inhibitor. The increase in the levels of the peptide may reflect the decrease in the β5-subunit cleavage and compensatory increase in the usage of other sites for protein breakdown. While the changes in the levels of the 2–16 and 2–15 peptides fit the expected result due to bortezomib inhibition of the β5-subunit, the changes in the other N-terminal fragments of this protein do not fit into this hypothesis. Specifically, the 2–14, 2–10, and 2–9 peptides show statistically significant increases upon treatment with 50 or 500 nM bortezomib for all time points examined, and for some peptides, also with 5 nM drug for 16 hours (Figure 4A and B). The 2–14 peptide requires cleavage at an Asp, which should be mediated by the β1-subunit; this activity is substantially inhibited by 500 nM bortezomib, and partially by 50 nM of this drug [45]. Furthermore, both the 2–9 and 2–10 peptides contain hydrophobic residues on their C-termini; these would require β5-proteasome activity and therefore should be reduced in the presence of bortezomib.

**Figure 5:**
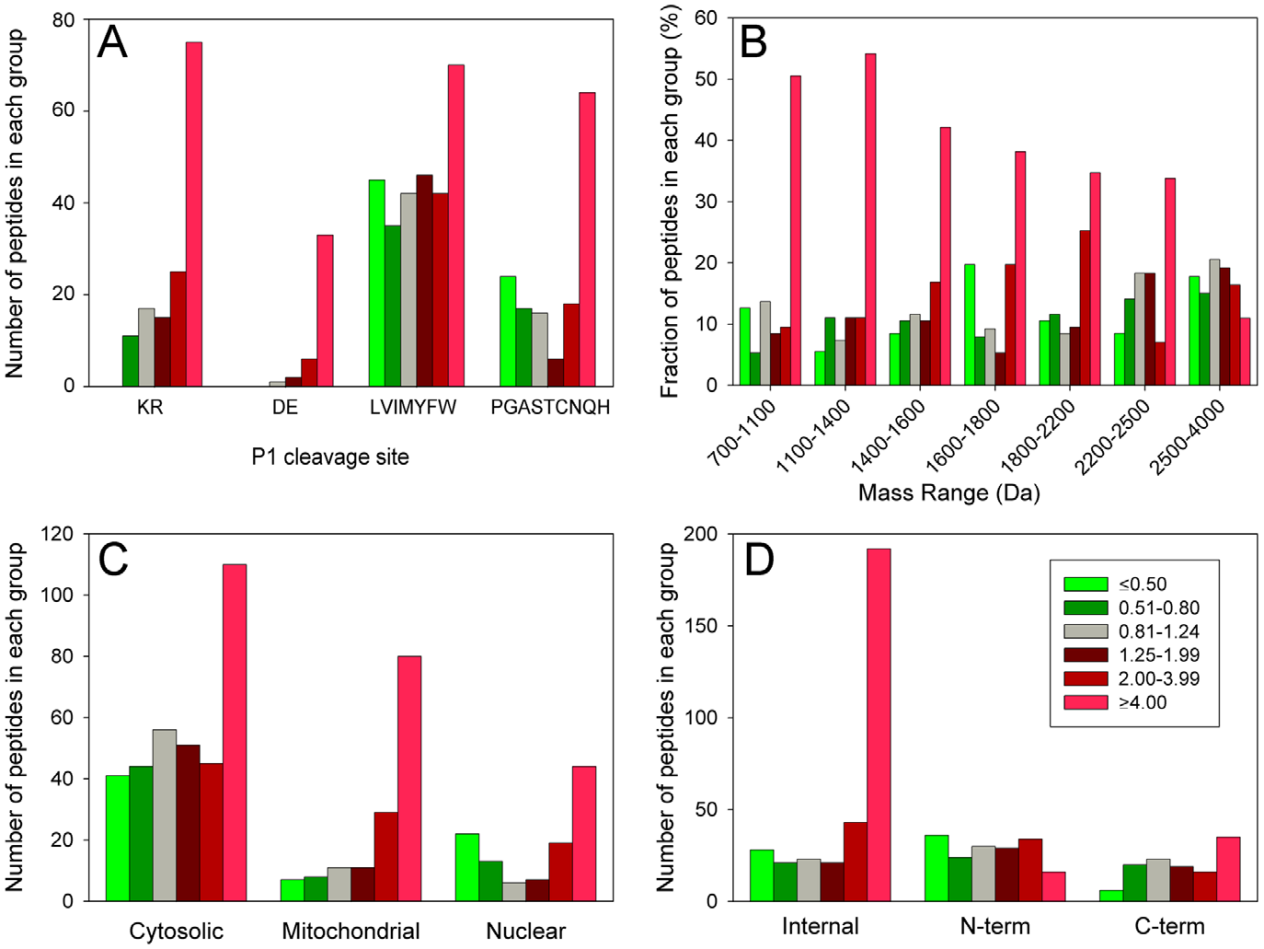
Analysis of identified peptides. Three experiments were considered for this analysis: HEK293T cells treated for 1 hour with 50 or 500 nM bortezomib, and SH-SY5Y cells treated for 1 hour with 500 nM bortezomib. The average of the three biological replicates in each experiment was grouped into the ranges shown in the inset to panel D; this corresponds to the ranges used in previous figures. Peptides detected in each of the three experiments were considered separately, resulting in 642 peptides for this analysis. A: Correlation of the effect of bortezomib with cleavage site residue. The amino acid in the P1 site required to generate the peptide was considered. For peptides representing internal fragments of the proteins, two cleavage sites are needed to generate the peptide, and if either side was an acidic residue (D or E), it was considered in this group. For the remaining peptides, if either side was a basic residue (K or R), it was considered in this group. Finally, for the remaining peptides, if both sides were a hydrophobic residue (LVIMYFW), it was considered in this group. The fourth group (PGASTCNQH) represents peptides in none of the previous groups. B: Correlation of the effect of bortezomib with peptide mass. For this analysis, the mass ranges were chosen to provide ∼100 peptides in each group, and the y-axis was adjusted to the fraction of peptides in each group and not the total number as plotted in the other panels. C: Correlation of the effect of bortezomib with intracellular location of the precursor protein, determined from literature searches. This analysis excluded a small number of proteins that did not have a well characterized intracellular location (see Table S1). D: Correlation of the effect of bortezomib with the location of the peptide within the protein precursor. The N-terminal group includes peptides lacking the initiation Met.

To extend the cleavage site analysis to a larger number of peptides, we combined all of the data from the experiments testing 50 and 500 nM bortezomib on both HEK293T and SH-SY5Y cells for the 1 hour time points. For each experiment, the average level of peptide in the replicates was used (as done for Figure 4), rather than each individual group value (as shown in Figures 2 and 3). Then, we sorted all identified peptides by the P1 residue of the cleavage site necessary to generate the peptide and determined the number of peptides with ratios in each of the 6 previously defined groups (i.e. ≤0.50; 0.51–0.80, etc). As found for the heat shock 10kDa protein 1 fragments shown in Figure 4, a large number of other peptides that require cleavage by the β1 (Asp, Glu) or β2 proteasome (Lys, Arg) are greatly elevated upon bortezomib treatment (Figure 5A). None of the peptides representing β1 or β2 cleavages were greatly decreased by bortezomib treatment, whereas many of the β5 cleavages (hydrophobic residues) were greatly decreased (Figure 5A). Still, a large number of the β5 cleavages were also greatly elevated by the bortezomib treatment (Figure 5A), as found for some of the heat shock 10kDa protein 1 fragments shown in Figure 4.

A similar analysis was performed in order to test if the changes in peptide levels correlated with peptide mass. The prediction was that in the presence of the proteasome inhibitor, protein cleavage would be less complete and this would result in larger peptides. However, the observed change was the opposite of the prediction; approximately 50% of the highly-elevated peptides were in the 700–1100 Da and 1100–1400 Da groups (Figure 5B). In contrast, only 30–35% of the peptides in the 1800–2200 and 2200–2500 Da groups, and 12% in the 2500–4000 Da groups were greatly elevated by bortezomib.

Previously, it was noted that many of the observed cellular peptides were derived from cytosolic proteins, although peptides were also found that corresponded to mitochondrial and nuclear proteins. To test if the cellular location of the protein correlated with changes in peptides, the number of peptides in each group were compared (Figure 5C). As previously found, most of the peptides detected in the present study were derived from cytosolic proteins. Over 100 of these cytosolic protein-derived peptides were greatly elevated by bortezomib treatment, but this represented only ∼30% of the total number of cytosolic protein-derived peptides, and many peptides derived from cytosolic proteins showed no change or decreased upon treatment (Figure 5C). In contrast, the majority of the mitochondrial protein-derived peptides showed a very large increase upon bortezomib treatment, and only a handful did not change or showed a decrease (Figure 5).

**Figure 6:**
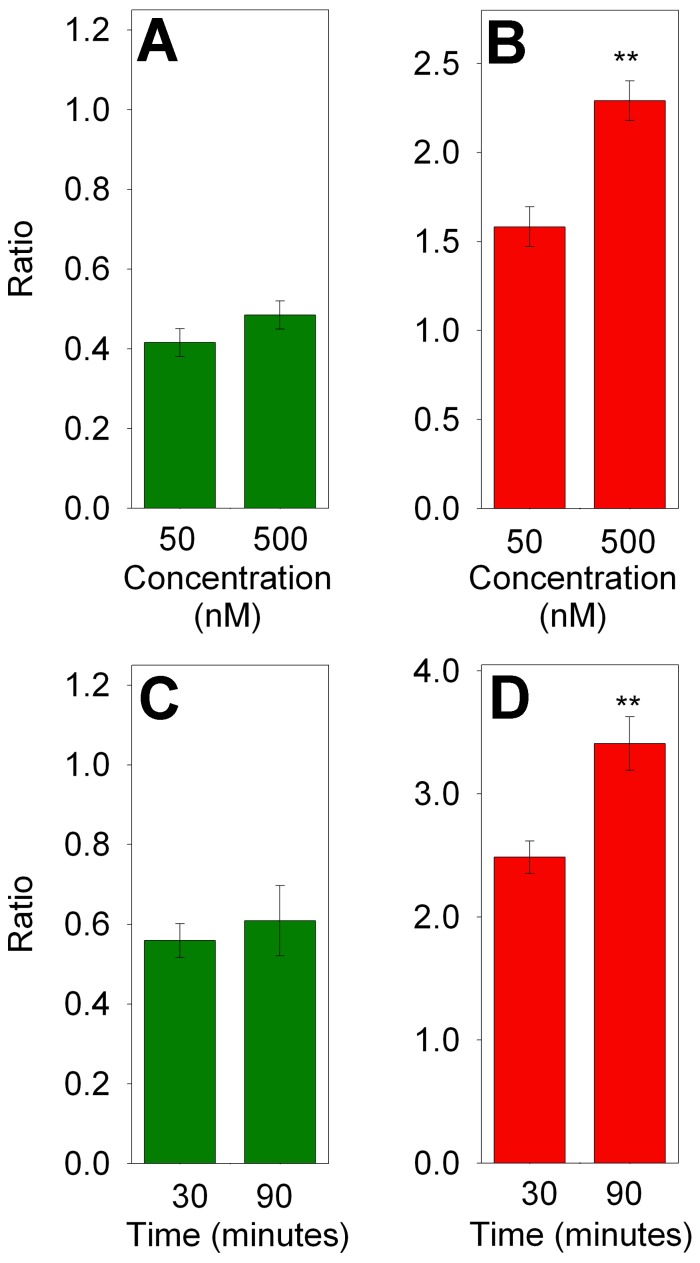
Dose-dependence and time-dependence of peptides that showed partial decreases (left panels) and increases (right panels) in response to bortezomib. A,B: HEK293T cells were treated for 1 hour with the indicated concentration of bortezomib. Peptides that partially decreased (ratio 0.2 to 0.8, n = 26) or partially increased (ratio 1.25 to 4.0, n = 50) in the 500 nM data set were compared to the same peptides observed in the 50 nM data set. C, D: HEK293T cells were treated for the indicated time with 500 nM bortezomib. Peptides that partially decreased (n = 13) or increased (n = 20) in the 30 minute group were compared to the same peptides in the 90 minute group. **, p<0.01 using Student’s t-test. Data used for these analyses are included in supplemental Tables S3 and S4.

In previous studies, approximately 50% of the cellular peptides in HEK293T and other cell lines were found to represent the N- or C-terminus of the protein [30]. In the present study, approximately 70% of peptides unaffected by bortezomib were N- or C-terminal peptides and only 30% represented internal peptides (Figure 5D, grey bars). In contrast, ∼80% of the peptides which were greatly elevated by bortezomib represented internal fragments of the proteins (Figure 5D, bright red bars). For the analysis shown in Figure 5, both 50 and 500 nM treatments were combined. To determine if the peptides that showed a partial decrease or increase were comparable between these two concentrations of bortezomib, the two groups were analyzed separately. For this analysis, only those peptides detected in both the 50 and 500 nM treatment groups were considered. The 26 peptides which showed a partial decrease (ratio 0.20 to 0.80) in the 500 nM bortezomib group were similarly affected by treatment with 50 nM bortezomib (Figure 6A), indicating that the partial effect was not due to incomplete inhibition of bortezomib at the lower dose. However, those peptides partially increased by 50 nM bortezomib showed a significantly larger increase upon treatment with 500 nM drug (Figure 6B). Similar analysis was performed to compare peptides that partially decreased or increased upon treatment with 500 nM bortezomib for 30 or 90 minutes. Levels of peptides that partially decreased after 30 minutes of treatment were not significantly different after 90 minutes of treatment (Figure 6C), indicating that the partial decrease was not due to incomplete inhibition at the earlier time point. However, peptides that partially increased after 30 minutes of bortezomib treatment showed a significantly greater increase after 90 minutes of treatment. Thus, the bortezomib-induced increase in peptides showed a dose- and time-dependence increase, whereas the drug-induced partial decrease in peptides was not influenced by concentration of drug or length of treatment.

**Figure 7:**
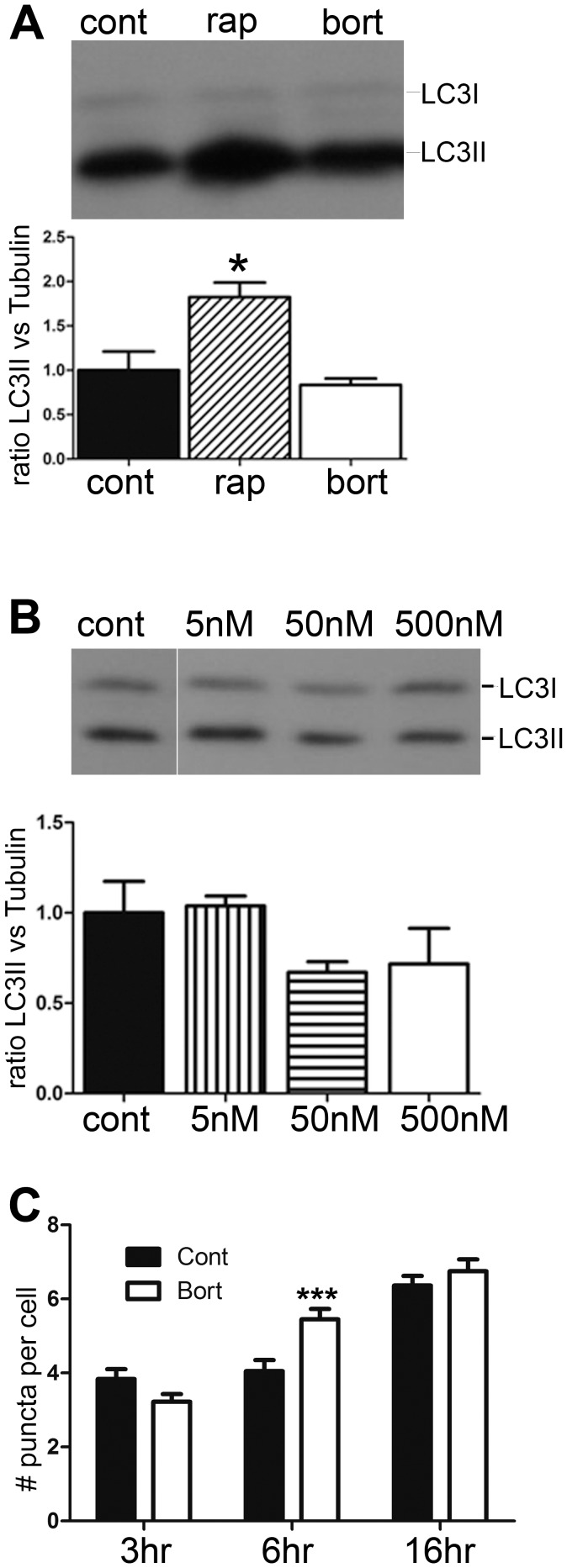
Analysis of autophagy in cells treated with bortezomib. HEK293T cells were treated with different concentrations of bortezomib and the levels of autophagy were analyzed by Western blotting and immunocytochemistry. A, Upper panel: Representative western blot for LC3 protein in HEK293T cells after treatment with 250 nM rapamicin (rap) or 500 nM bortezomib (bort) for 1 hour, compared to control cells (cont). Lower panel: Densitometric analysis of the levels of LC3II protein in control and treated HEK293T cells. The LC3II band densities were normalized with the corresponding α-tubulin bands. Band densities were measured using the Odyssey Infrared Imaging System. Error bars represent standard error of the mean (n  = 4). *p<0.05 using Student’s t-test. B, Upper panel: Representative western blots for LC3 protein after treatment of HEK293T cells with the indicated concentrations of bortezomib for 1 hour. Lower panel: Densitometric analysis of the levels of LC3II protein in control and treated HEK293T cells, relative to α-tubulin. Error bars represent standard error of the mean (n  = 3). *p<0.05 using Student’s t-test. C: Number of LC3 puncta in HEK293T cells after treatment with 5 nM bortezomib for 3, 6, and 16 hours. Treated cells were subjected to immunostaining with antibodies against LC3. Number of puncta within each cell was counted by an observer who was blinded to the treatment. At least 150 cells were analyzed for each treatment condition.

The increase in peptides, especially internal peptides derived from mitochondrial proteins was unexpected. One possible mechanism would be through elevated autophagy [46]–[49]. To investigate if bortezomib treatments induce autophagy under the conditions that affected cellular peptide levels, we examined the forms of LC3 by Western blot analysis after treating cells with bortezomib. In one experiment, SH-SY5Y cells were treated with 500 nM bortezomib for 1 hour, with 250 nM rapamycin for 1 hour, or incubated with DMSO. While rapamycin caused a significant increase in the level of LC3II, bortezomib had no effect (Figure 7A). HEK293T cells were also examined in this assay, and the effect of 5, 50, and 500 nM bortezomib tested for 1 hour. None of the concentrations of bortezomib caused an increase in LC3II, indicating the absence of autophagy (Figure 7B). We also tested the effect of long-term treatments with 5 nM bortezomib on HEK293T cells, using an assay that involved immunofluorescence to detect LC3 and counting of the number of puncta per cell. Neither the 3 hr nor the 16 hr time points showed a statistically significant difference in the number of puncta between the bortezomib-treated and control cells (Figure 7C). However, cells treated with 5 nM bortezomib for 6 hours showed a small statistically significant increase in the number of autophagic puncta (Figure 7C). Therefore, while the 6 hour time point induced autophagy, other time points used for the peptidomics analysis did not.

**Figure 8:**
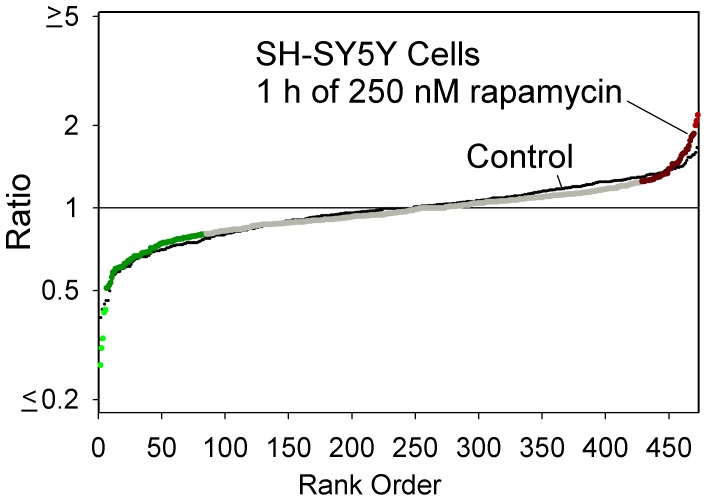
Peptidomics summary of SH-SY5Y cells after induction of autophagy. Cells were treated with 250 nM rapamycin for 1 hour to induce autophagy and analyzed by the quantitative peptidomics technique. The data are plotted using the approach shown in Figure 2. Large colored circles represent the rapamycin-treated samples, relative to the average control value, while the small black circles represent each individual control replicate compared to the average control value.

Because there was a slight induction of autophagy at one of the time points, we tested if autophagy could induce a change in peptide levels and therefore contribute to the observed results. For this, SH-SY5Y cells were treated with 250 nM rapamycin for 1 hour and then analyzed by peptidomics. A plot of the ratio for all observed peptides (identified and unidentified) shows no major difference between the rapamycin-treated replicates and the control replicates (Figure 8). Therefore, it is unlikely that autophagy contributes to the changes in intracellular peptide levels observed upon bortezomib treatment.

## Discussion

Because bortezomib is a clinically useful drug, it is important to understand the effects of the drug at a molecular level. Previous studies have focused on the effect of bortezomib on protein turnover, and the present study is the first to examine the products of protein turnover–the peptides. There are two major findings of the present study. The finding that levels of some peptides are reduced by treatment with bortezomib supports the hypothesis that the proteasome is responsible for their production, as predicted from a recent study examining the effect of epoxomicin on peptide levels [29]. The finding that the majority of peptides detected in the present study are elevated by treatment with bortezomib was unexpected. There are four possible explanations, all of which may contribute in part. These possibilities are further discussed below.

One likely explanation for the increased levels of some peptides is that bortezomib blocks the major processing activities and allows minor pathways to contribute to a greater extent. Because bortezomib is known to inhibit the β1 and β5 activities of the proteasome, but not the β2 activity, it would be expected that cleavages at hydrophobic and acidic residues would decrease while those at basic residues would either be unaffected or increase as a result of the blockade of the other activities. Consistent with this, bortezomib was previously found to stimulate the β2 activity of the proteasome [32], [34], [50], [51]. But, this explanation can only account for the increased levels of peptides that are produced by the β2 proteasome subunit. Based on analysis of the P1 residue of the cleavage site required to generate the peptides (Figure 5A), less than half of the peptides found to greatly increase in the present study arise from β2 proteasome activity. Instead, many of the peptides that increased upon bortezomib treatment contained hydrophobic or acidic residues in the P1 site. Unless the β2 activity is able to generate these peptides, an increase in the β2 activity would be unlikely to account for their increased levels.

A second explanation for the observed increase in many intracellular peptides is the possibility that bortezomib activates another cellular protease system. Bortezomib is known to activate caspases [35]. However, most of the peptides that increased in the HEK293T cells in response to bortezomib are not produced by caspases based on analysis of the cleavage sites; only a small number contain an Asp in the P1 position, and the vast majority of the cleavage sites required to generate the observed peptides do not match the caspase consensus site. It is also possible that calpain-mediated cleavage of proteins is elevated by bortezomib; this has been proposed to explain the increased degradation of IκBα caused by bortezomib treatment of various human cell lines [52]. However, a previous peptidomics study did not detect major changes in levels of intracellular peptides when SH-SY5Y cells were treated with a calcium ionophore known to activate calpains [30]. Another possibility is activation of autophagy by bortezomib, which is known to induce autophagy in several systems [46]–[49]. This idea is attractive because of the large number of mitochondrial protein fragments found to be elevated by bortezomib (Figure 5C). However, the standard marker for autophagy, LC3, showed no evidence of autophagy upon treatment of HEK293T or SH-SY5Y cells with high concentrations of bortezomib for 1 hour (Figure 7). Furthermore, treatment of cells with rapamycin for 1 hour to produce autophagy had little effect on the cellular peptides (Figure 8). Taken together, these results suggest that autophagy does not contribute to the altered peptidome observed upon treatment of cells with bortezomib for short time periods.

A third possibility to explain the increase in many peptides is that protein levels are induced by bortezomib. A previous RNA microarray analysis found that thousands of mRNAs were either up- or down-regulated by treatment with bortezomib for 14, 24, or 48 hours [53]. A cross-comparison of the RNA microarray study and our results found no correlation between those proteins corresponding to up-regulated peptides and mRNA changes at 14 and 24 hour time points. Furthermore, our finding that most of the peptides were up-regulated as early as 30 minutes after the start of the exposure to bortezomib argues against a general effect on protein synthesis; while the synthesis of some proteins may be stimulated within 30 minutes of the start of bortezomib treatment, it is unlikely that all of the affected proteins will show such a rapid increase. Furthermore, a change in protein levels would not explain why all peptides derived from a particular protein are not similarly affected. For example, although many fragments of heat shock 10 kDa protein 1 (chaperonin 10) are elevated, one fragment is significantly decreased by 50 and 500 nM bortezomib (Figures 3 and 4). Similar variability in the changes of peptides derived from other proteins is observed (Figure 3). Therefore, an increase in the synthesis of these proteins would not explain why some of the peptides derived from these proteins were decreased by the bortezomib treatment.

A fourth possibility is that bortezomib interferes with the further degradation of the peptides produced by the proteasome. Although bortezomib is usually described in the literature as being highly specific for the proteasome with no off-target effects, a recent study has shown that bortezomib inhibits serine proteases such as cathepsins A and G, chymase, dipeptidyl peptidase II, and HtrA2/Omi [54]. Although none of these enzymes are thought to function in the degradation of peptides produced by the proteasome, it is possible that bortezomib has additional off target effects and inhibits a cytosolic peptidase. This possibility would be consistent with the increase in peptides derived from cytosolic proteins as well as mitochondrial proteins; this organelle contains transporters that export peptides generated by proteases within the mitochondria, and these peptides are subsequently degraded by cytosolic peptidases.

The mechanism of action of bortezomib as an antitumor agent is thought to involve a reduction in protein turnover and/or protein activation, especially for proteins such as NFκB and cyclins [55], [56]. To generate the active NFκB transcriptional dimeric complexes, two precursors, NFκB1 (p105) and NFκB2 (p100), have to undergo limited proteolytic processing by the proteasome to yield the respective shorter active subunits p50 and p52 that represent the N-terminal domains of their precursors, whereas the C-terminal segments are degraded following processing [57]. However, high concentrations of bortezomib are required to produce only modest changes in protein levels [45]. In contrast, bortezomib causes dramatic changes in the cellular peptidome. Although intracellular peptides are generally considered to be inactive protein fragments that are in the process of degradation, many studies have found that synthetic peptides of 10–20 amino acids can affect protein-protein interactions [7]–[9], [58]. Thus, the endogenous peptides identified in this study, as well as in numerous other peptidomics studies, may have cellular functions. If these cytosolic peptides are functional, then the bortezomib-induced change in the peptide profile would likely have physiological effects that contribute to the drug’s anticancer action and/or side effects. Interestingly, when the 48 proteins that give rise to the majority of peptides altered by bortezomib treatment of HEK293T cells (i.e. those listed in Table S2) were subjected to pathway analysis using the Ingenuity System program, all 48 of these proteins were grouped into a single network that functions in cell growth, proliferation, and death (Figure S3). Thus, the changes in peptides derived from these proteins may reflect altered degradation of these proteins and/or increased stability of peptides that function in modulating protein-protein interactions.

## Supporting Information

Figure S1 Strategy of treatment and peptidomics analysis. Top: Labeling scheme for a typical experiment. HEK293T or SH-SY5Y cells were grown in 150 mm plates to ∼90% confluency in Dulbecco's modified Eagle's medium containing 10% fetal bovine serum. In a typical experiment, 2–3 plates of cells were used for each treatment group replicate. Three replicates were treated with bortezomib (diluted from a 10 mM stock in DMSO) and two replicates were treated without drug but with the same small amount of DMSO as in the bortezomib groups. Following incubation, media were removed, cells were extensively washed with PBS, and the peptides were extracted and labeled with TMAB-NHS isotopic tags. A typical scheme involved labeling of the bortezomib-treated peptides with D0, D6, and D12-TMAB-NHS and control cells with D3 and D9-TMAB-NHS. In other experiments, the bortezomib-treated and control groups were labeled differentially (controls were D0 and D12 in some experiments, and D9 and D12 in other experiments such as those shown in Figure 4). Following the labeling with the isotopic reagent and quenching of the unreacted label, the samples were pooled, the peptides were further purified, and then analyzed by LC/MS. Bottom: Representative results, and quantitative approach. The MS spectra for the peptide subsequently identified by MS/MS analysis as the heat shock 10kDa protein 1 (chaperonin 10) N-terminal 2–10 peptide (AcAGQAFRKFL) with 1 TMAB tag and one proton (2+ charge), from the experiment testing 50 nM bortezomib for 1 hour. The peak intensity was determined for each of the 5 TMAB peaks from the monoisotopic peak and the peak containing one ^13^C atom (red arrows). For those peptides without base-line separation between the TMAB groups (as in this example), the background signal from the lower mass peak was subtracted (blue horizontal lines). This was determined from isotopic distribution of ^13^C in each peptide. For the peptide in this example, the peak with 3 atoms of ^13^C is ∼15% of the signal of the monoisotopic peak, and the peak with 4 atoms is ∼7% of the monoisotopic peak. Red arrows show the calculated peak intensities after subtraction of the baseline. For peptides labeled with 2 or more TMAB tags, this procedure was unnecessary due to baseline separation of the signals.
(PDF)

Figure S2 Inhibition of HEK293T cell proteasome activity with bortezomib. HEK293T cells were lysed by sonication in 50 mM Tris HCl buffer, pH 7.5, containing 40 mM KCl, 5 mM MgCl_2_, 0.5 mM ATP, and 1 mM DTT. Dilutions were tested for optimal activity using 100 µM final concentration of the proteasome substrate succinyl-Leu-Leu-Val-Tyr-7-amino-4-methylcoumarin (Succ-LLVY-AMC) in 200 µl of homogenization buffer and incubation for 1 hour at 37°C. Product was detected by dilution of the enzyme reaction into 2 ml of ice-cold 50 mM Tris HCl, pH 7.5, and measurement of fluorescence (380 nm excitation, 460 nm emission). A dilution of cell extract that provided ∼5% cleavage of the substrate into product over the incubation time was used for the assay with bortezomib, which was preincubated with extract for 10 minutes at 25°C prior to addition of substrate and incubation for 1 hour at 37°C. Error bars show standard error of the mean (n = 3); data points without error bars had error ranges smaller than the symbol size. **, p<0.01 versus the ‘no inhibitor’ control, using Student’s t-test.
(PDF)

Figure S3 Interactome of proteins. The 48 distinct proteins that give rise to the peptides reported in the heat map (Figure 3 and Table S2) were subjected to Ingenuity Pathway Analysis (Ingenuity Systems, Inc, version 12710793, 2012-05-08). Networks were algorithmically generated based on their connectivity. Molecules are represented as nodes, and the biological relationship between two nodes is represented as a line (solid lines indicate direct interactions, dashed lines represent indirect interactions). All relationships are supported by at least one reference from the literature, from a textbook, or from canonical information stored in the Ingenuity Pathways database. Human, mouse, and rat orthologs of a gene are stored as separate objects in the Ingenuity Pathways database, but are represented as a single node in the network. Nodes are displayed using various shapes that represent the functional class of the gene product. Filled nodes (grey) represent the proteins identified in the data set and unfilled nodes represent proteins that are part of the network but which were not identified in the present study. The network shown in the figure is the merged composite of three primary networks, each of which is related. Network 1 is involved in cell death, cellular growth and proliferation, and free radical scavenging and contains the following gene products: C1QBP, CD3, Ck2, CLNS1A, COX5A, COX7C, EEF1B2, EIF5A, FKBP1A, FUBP1, HINT1, HIST2, H2BE, HISTONE, HNRNPA2B1, Ikb, NFkB, NME2, NPM1, PARK7, PEBP1, PHB, PPIA, PRDX5, RBM3, Ribosomal 40s subunit, Rnr, RPS12, RPS21, RPS28, SET, SNRPG, SRSF1, SRSF2, TXN, and VIM. Network 2 is involved in free radical scavenging, molecular transport, and cancer and contains the following gene products: 60S ribosomal subunit, APP, ARRB2, COX6B1, COX7B2, COX8C, Cytochrome c oxidase, ERBB2, ERH, Gm5619/Gm5845, HN1, LOC342994, LOC646875, LOC100360491, LOC100361259, MAPK1IP1L, miR-124, MYC, MYCN, NDUFA8, NDUFAB1, PFDN1, RPL22L1, Rpl22l2, RPL26L1, RPL36AL, Rpl38, RPL39L, RPL7L1, RPLP1, RPLP2, SOD1, TMSB10/TMSB4X, TPI1, and UBA52. Network 3 functions in cellular growth and proliferation, endocrine system development and function, and protein synthesis, and includes the following gene products: Akt, ANGPTL1, CDKN1B, Ctla2a, DNAJB7, DUT, Dut, ERK, ERK1/2, GAST, GCNT2, HNRPDL, HRSP12, HSPE1, IFNG, IL12, IL12/23R, IL12B, INSRR, Insulin, KHSRP, KLRB1, Mcpt8, MIF, miR-376a/miR-376b/miR-376b-3p, NUTF2, Pkc(s), POMP, PPARA, SLC38A3, SLC5A2, SUMO4, TLR3/4, and VTCN1.
(PDF)

Table S1 Summary of peptidomic results from all experiments involving bortezomib, epoxomicin, or rapamycin treatment of HEK293T or SH-SY5Y cells. Abbreviations: z, charge; T, number of TMAB tags; Theor. mass, theoretical monoisotopic mass; Adj Up, adjacent upstream amino acid residue in precursor; Adj Dwn, adjacent downstream amino acid residue in precursor; *, terminus of protein; Mox, oxidized Met; Ac, acetyl. Sequences in parenthesis were only tentatively identified; MS/MS was not sufficient for conclusive identification
(XLSX)

Table S2 Heatmap data. This table shows the data used to generate Figure 3, and indicates gene name, protein name, sequence of peptide and theoretical mass (Theor. Mass), the location of the protein within the cell, the upstream and downstream cleavage sites (including P1 and P1’ residues), and prediction of which proteasome subunit is involved in the generation of the peptide.
(XLSX)

Table S3 Dose response of peptides that showed partial decreases and increases in response to bortezomib. This table shows the data used to plot Figure 6 A and B. Abbreviations are defined in Table S1. Additional abbreviations: Adj Up, residue adjacent to the N-terminal side of the peptide; Adj Dwn, residue adjacent to the C-terminal side of the peptide. All masses are monoisotopic.
(XLSX)

Table S4 Time-dependence of peptides that showed partial decreases and increases in response to bortezomib. This table shows the data used to plot Figure 6 C and D. Abbreviations as in Tables S1 and S3.
(XLSX)
