# Supplementary material for: Alterations of the Intracellular Peptidome in Response to the Proteasome Inhibitor Bortezomib
Source: PLoS One. 2013 Jan 7;8(1):e53263. doi: 10.1371/journal.pone.0053263 (PMC3538785; doi:10.1371/journal.pone.0053263)
Supplement: Figure S1 — Strategy of treatment and peptidomics analysis. Top: Labeling scheme for a typical experiment. HEK293T or SH-SY5Y cells were grown in 150 mm plates to ∼90% confluency in Dulbecco's modified Eagle's medium containing 10% fetal bovine serum. In a typical experiment, 2–3 plates of cells were used for each treatment group replicate. Three replicates were treated with bortezomib (diluted from a 10 mM stock in DMSO) and two replicates were treated without drug but with the same small amount of DMSO as in the bortezomib groups. Following incubation, media were removed, cells were extensively washed with PBS, and the peptides were extracted and labeled with TMAB-NHS isotopic tags. A typical scheme involved labeling of the bortezomib-treated peptides with D0, D6, and D12-TMAB-NHS and control cells with D3 and D9-TMAB-NHS. In other experiments, the bortezomib-treated and control groups were labeled differentially (controls were D0 and D12 in some experiments, and D9 and D12 in other experiments such as those shown in Figure 4). Following the labeling with the isotopic reagent and quenching of the unreacted label, the samples were pooled, the peptides were further purified, and then analyzed by LC/MS. Bottom: Representative results, and quantitative approach. The MS spectra for the peptide subsequently identified by MS/MS analysis as the heat shock 10kDa protein 1 (chaperonin 10) N-terminal 2–10 peptide (AcAGQAFRKFL) with 1 TMAB tag and one proton (2+ charge), from the experiment testing 50 nM bortezomib for 1 hour. The peak intensity was determined for each of the 5 TMAB peaks from the monoisotopic peak and the peak containing one 13C atom (red arrows). For those peptides without base-line separation between the TMAB groups (as in this example), the background signal from the lower mass peak was subtracted (blue horizontal lines). This was determined from isotopic distribution of 13C in each peptide. For the peptide in this example, the peak with 3 atoms of 13C is ∼15% of the [file pone.0053263.s001.pdf]

Supplemental Material: Alterations of the intracellular peptidome in response to the proteasome inhibitor bortezomib

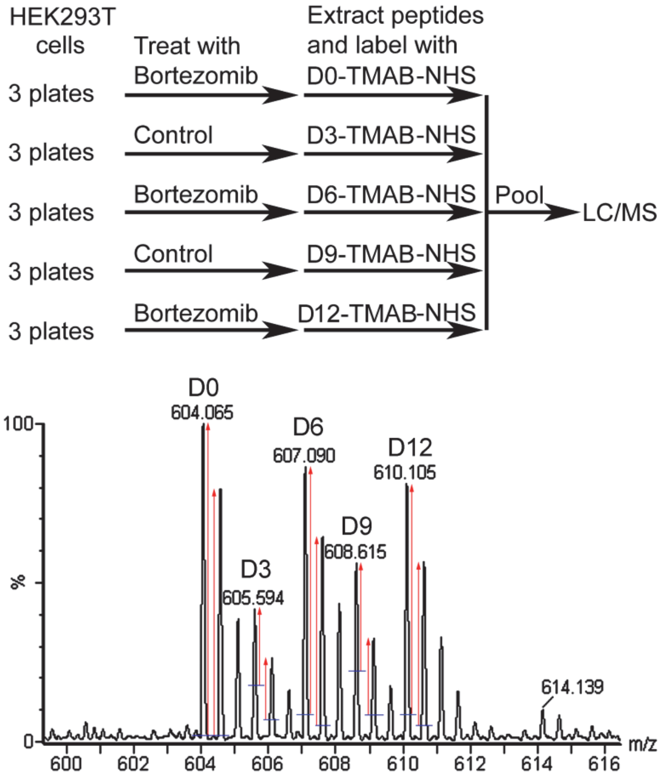

	B1	C1	B2	C2	B3
Monoisotopic	41	10	33	14	31
13C-peak	32	8	25	10	22
Total intensity	73	18	58	24	53
Relative to average control	3.47	0.86	2.76	1.14	2.52

Figure S1. Strategy of treatment and peptidomics analysis. Top: Labeling scheme for a typical experiment. HEK293T or SH-SY5Y cells were grown in 150 mm plates to ~90% confluency in Dulbecco's modified Eagle's medium containing 10% fetal bovine serum. In a typical experiment, 2-3 plates of cells were used for each treatment group replicate. Three replicates were treated with bortezomib (diluted from a 10 mM stock in DMSO) and two replicates were treated without drug but with the same small amount of DMSO as in the bortezomib groups. Following incubation, media were removed, cells were extensively washed with PBS, and the peptides were extracted and labeled with TMAB-NHS isotopic tags. A typical scheme involved labeling of the bortezomib-treated peptides with D0, D6, and D12-TMAB-NHS and control cells with D3 and D9-TMAB-NHS. In other experiments, the bortezomib-treated and control groups were labeled differentially (controls were D0 and D12 in some experiments, and D9 and D12 in other experiments such as those shown in Figure 4). Following the labeling with the isotopic reagent and quenching of the unreacted label, the samples were pooled, the peptides were further purified, and then analyzed by LC/MS. Bottom: Representative results, and quantitative approach. The MS spectra for the peptide subsequently identified by MS/MS analysis as the heat shock 10kDa protein 1 (chaperonin 10) N-terminal 2-10 peptide (AcAGQAFRKFL) with 1 TMAB tag and one proton (2+ charge), from the experiment testing 50 nM bortezomib for 1 hour. The peak intensity was determined for each of the 5 TMAB peaks from the monoisotopic peak and the peak containing one ^{13}C atom (red arrows). For those peptides without base-line separation between the TMAB groups (as in this example), the background signal from the lower mass peak was subtracted (blue horizontal lines). This was determined from isotopic distribution of ^{13}C in each peptide. For the peptide in this example, the peak with 3 atoms of ^{13}C is ~15% of the signal of the monoisotopic peak, and the peak with 4 atoms is ~7% of the monoisotopic peak. Red arrows show the calculated peak intensities after subtraction of the baseline. For peptides labeled with 2 or more TMAB tags, this procedure was unnecessary due to baseline separation of the signals.
