# Supplementary material for: Alterations of the Intracellular Peptidome in Response to the Proteasome Inhibitor Bortezomib
Source: PLoS One. 2013 Jan 7;8(1):e53263. doi: 10.1371/journal.pone.0053263 (PMC3538785; doi:10.1371/journal.pone.0053263)
Supplement: Figure S2 — Inhibition of HEK293T cell proteasome activity with bortezomib. HEK293T cells were lysed by sonication in 50 mM Tris HCl buffer, pH 7.5, containing 40 mM KCl, 5 mM MgCl2, 0.5 mM ATP, and 1 mM DTT. Dilutions were tested for optimal activity using 100 µM final concentration of the proteasome substrate succinyl-Leu-Leu-Val-Tyr-7-amino-4-methylcoumarin (Succ-LLVY-AMC) in 200 µl of homogenization buffer and incubation for 1 hour at 37°C. Product was detected by dilution of the enzyme reaction into 2 ml of ice-cold 50 mM Tris HCl, pH 7.5, and measurement of fluorescence (380 nm excitation, 460 nm emission). A dilution of cell extract that provided ∼5% cleavage of the substrate into product over the incubation time was used for the assay with bortezomib, which was preincubated with extract for 10 minutes at 25°C prior to addition of substrate and incubation for 1 hour at 37°C. Error bars show standard error of the mean (n = 3); data points without error bars had error ranges smaller than the symbol size. **, p<0.01 versus the ‘no inhibitor’ control, using Student’s t-test. (PDF) [file pone.0053263.s002.pdf]

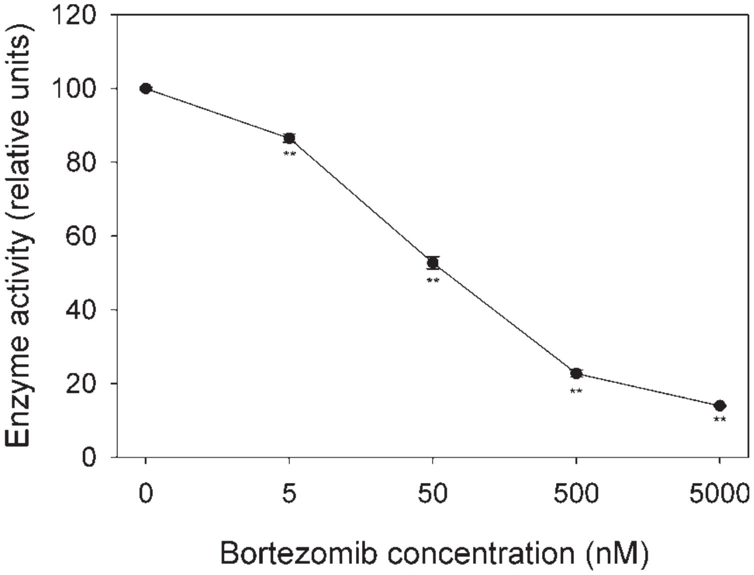

Figure S2. Inhibition of HEK293T cell proteasome activity with bortezomib. HEK293T cells were lysed by sonication in 50 mM Tris HCl buffer, pH 7.5, containing 40 mM KCl, 5 mM $MgCl_2$, 0.5 mM ATP, and 1 mM DTT. Dilutions were tested for optimal activity using 100 µM final concentration of the proteasome substrate succinyl-Leu-Leu-Val-Tyr-7-amino-4-methylcoumarin (Succ-LLVY-AMC) in 200 µl of homogenization buffer and incubation for 1 hour at 37°C. Product was detected by dilution of the enzyme reaction into 2 ml of ice-cold 50 mM Tris HCl, pH 7.5, and measurement of fluorescence (380 nm excitation, 460 nm emission). A dilution of cell extract that provided ~5% cleavage of the substrate into product over the incubation time was used for the assay with bortezomib, which was preincubated with extract for 10 minutes at 25°C prior to addition of substrate and incubation for 1 hour at 37°C. Error bars show standard error of the mean (n=3); data points without error bars had error ranges smaller than the symbol size. **, $p<0.01$ versus the 'no inhibitor' control, using Student's t-test.
