# Supplementary material for: Alterations of the Intracellular Peptidome in Response to the Proteasome Inhibitor Bortezomib
Source: PLoS One. 2013 Jan 7;8(1):e53263. doi: 10.1371/journal.pone.0053263 (PMC3538785; doi:10.1371/journal.pone.0053263)
Supplement: Figure S3 — Interactome of proteins. The 48 distinct proteins that give rise to the peptides reported in the heat map (Figure 3 and Table S2) were subjected to Ingenuity Pathway Analysis (Ingenuity Systems, Inc, version 12710793, 2012-05-08). Networks were algorithmically generated based on their connectivity. Molecules are represented as nodes, and the biological relationship between two nodes is represented as a line (solid lines indicate direct interactions, dashed lines represent indirect interactions). All relationships are supported by at least one reference from the literature, from a textbook, or from canonical information stored in the Ingenuity Pathways database. Human, mouse, and rat orthologs of a gene are stored as separate objects in the Ingenuity Pathways database, but are represented as a single node in the network. Nodes are displayed using various shapes that represent the functional class of the gene product. Filled nodes (grey) represent the proteins identified in the data set and unfilled nodes represent proteins that are part of the network but which were not identified in the present study. The network shown in the figure is the merged composite of three primary networks, each of which is related. Network 1 is involved in cell death, cellular growth and proliferation, and free radical scavenging and contains the following gene products: C1QBP, CD3, Ck2, CLNS1A, COX5A, COX7C, EEF1B2, EIF5A, FKBP1A, FUBP1, HINT1, HIST2, H2BE, HISTONE, HNRNPA2B1, Ikb, NFkB, NME2, NPM1, PARK7, PEBP1, PHB, PPIA, PRDX5, RBM3, Ribosomal 40s subunit, Rnr, RPS12, RPS21, RPS28, SET, SNRPG, SRSF1, SRSF2, TXN, and VIM. Network 2 is involved in free radical scavenging, molecular transport, and cancer and contains the following gene products: 60S ribosomal subunit, APP, ARRB2, COX6B1, COX7B2, COX8C, Cytochrome c oxidase, ERBB2, ERH, Gm5619/Gm5845, HN1, LOC342994, LOC646875, LOC100360491, LOC100361259, MAPK1IP1L, miR-124, MYC, MYCN, NDUFA8, NDUFAB1, PFDN1, RPL22L1, Rpl22l2, RPL26L1, RPL [file pone.0053263.s003.pdf]

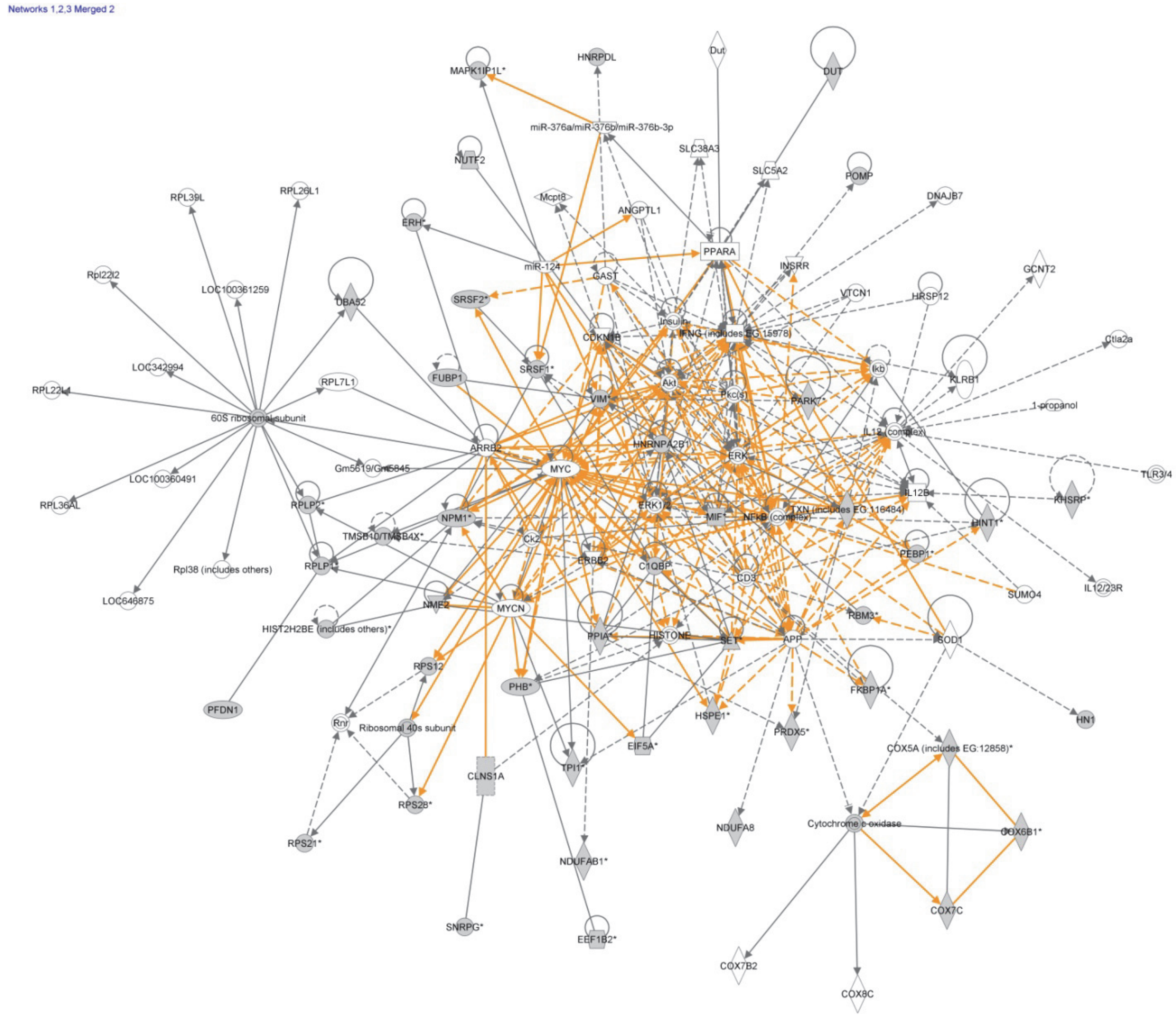

Figure S3: Interactome of proteins. The 48 distinct proteins that give rise to the peptides reported in the heat map (Figure 3 and Table S2) were subjected to Ingenuity Pathway Analysis (Ingenuity Systems, Inc, version 12710793, 2012-05-08). Networks were algorithmically generated based on their connectivity. Molecules are represented as nodes, and the biological relationship between two nodes is represented as a line (solid lines indicate direct interactions, dashed lines represent indirect interactions). All relationships are supported by at least one reference from the literature, from a textbook, or from canonical information stored in the Ingenuity Pathways database. Human, mouse, and rat orthologs of a gene are stored as separate objects in the Ingenuity Pathways database, but are represented as a single node in the network. Nodes are displayed using various shapes that represent the functional class of the gene product. Filled nodes (grey) represent the proteins identified in the data set and unfilled nodes represent proteins that are part of the network but which were not identified in the present study. The network shown in the figure is the merged composite of three primary networks, each of which is related. Network 1 is involved in cell death, cellular growth and

proliferation, and free radical scavenging and contains the following gene products: C1QBP, CD3, Ck2, CLNS1A, COX5A, COX7C, EEF1B2, EIF5A, FKBP1A, FUBP1, HINT1, HIST2, H2BE, HISTONE, HNRNPA2B1, Ikb, NFkB, NME2, NPM1, PARK7, PEBP1, PHB, PPIA, PRDX5, RBM3, Ribosomal 40s subunit, Rnr, RPS12, RPS21, RPS28, SET, SNRPG, SRSF1, SRSF2, TXN, and VIM. Network 2 is involved in free radical scavenging, molecular transport, and cancer and contains the following gene products: 60S ribosomal subunit, APP, ARRB2, COX6B1, COX7B2, COX8C, Cytochrome c oxidase, ERBB2, ERH, Gm5619/Gm5845, HN1, LOC342994, LOC646875, LOC100360491, LOC100361259, MAPK1IP1L, miR-124, MYC, MYCN, NDUFA8, NDUFAB1, PFDN1, RPL22L1, Rpl22l2, RPL26L1, RPL36AL, Rpl38, RPL39L, RPL7L1, RPLP1, RPLP2, SOD1, TMSB10/TMSB4X, TPI1, and UBA52. Network 3 functions in cellular growth and proliferation, endocrine system development and function, and protein synthesis, and includes the following gene products: Akt, ANGPTL1, CDKN1B, Ctla2a, DNAJB7, DUT, Dut, ERK, ERK1/2, GAST, GCNT2, HNRPDL, HRSP12, HSPE1, IFNG, IL12, IL12/23R, IL12B, INSRR, Insulin, KHSRP, KLRB1, Mcpt8, MIF, miR-376a/miR-376b/miR-376b-3p, NUTF2, Pkc(s), POMP, PPARA, SLC38A3, SLC5A2, SUMO4, TLR3/4, and VTCN1.
